# A methodology to ensure and improve accuracy of Ki67 labelling index estimation by automated digital image analysis in breast cancer tissue

**DOI:** 10.1186/bcr3639

**Published:** 2014-04-06

**Authors:** Arvydas Laurinavicius, Benoit Plancoulaine, Aida Laurinaviciene, Paulette Herlin, Raimundas Meskauskas, Indra Baltrusaityte, Justinas Besusparis, Darius Dasevicius, Nicolas Elie, Yasir Iqbal, Catherine Bor, Ian O Ellis

**Affiliations:** 1 Department of Pathology, Forensic Medicine and Pharmacology, Faculty of Medicine, Vilnius University, Vilnius, Lithuania; 2 National Center of Pathology, affiliate of Vilnius University Hospital Santariskiu Clinics, Vilnius, Lithuania; 3 Path-Image/BioTiCla, University of Caen, Caen, France; 4 Pathology Department, F. Baclesse Comprehensive Cancer Center, Caen, France; 5 Department of Histopathology, Molecular Medical Sciences, University of Nottingham, Nottingham, UK

## Abstract

**Introduction:**

Immunohistochemical Ki67 labelling index (Ki67 LI) reflects proliferative activity and is a potential prognostic/predictive marker of breast cancer. However, its clinical utility is hindered by the lack of standardized measurement methodologies. Besides tissue heterogeneity aspects, the key element of methodology remains accurate estimation of Ki67-stained/counterstained tumour cell profiles. We aimed to develop a methodology to ensure and improve accuracy of the digital image analysis (DIA) approach.

**Methods:**

Tissue microarrays (one 1-mm spot per patient, n = 164) from invasive ductal breast carcinoma were stained for Ki67 and scanned. Criterion standard (Ki67-Count) was obtained by counting positive and negative tumour cell profiles using a stereology grid overlaid on a spot image. DIA was performed with Aperio Genie/Nuclear algorithms. A bias was estimated by ANOVA, correlation and regression analyses. Calibration steps of the DIA by adjusting the algorithm settings were performed: first, by subjective DIA quality assessment (DIA-1), and second, to compensate the bias established (DIA-2). Visual estimate (Ki67-VE) on the same images was performed by five pathologists independently.

**Results:**

ANOVA revealed significant underestimation bias (*P* < 0.05) for DIA-0, DIA-1 and two pathologists’ VE, while DIA-2, VE-median and three other VEs were within the same range. Regression analyses revealed best accuracy for the DIA-2 (R-square = 0.90) exceeding that of VE-median, individual VEs and other DIA settings. Bidirectional bias for the DIA-2 with overestimation at low, and underestimation at high ends of the scale was detected. Measurement error correction by inverse regression was applied to improve DIA-2-based prediction of the Ki67-Count, in particular for the clinically relevant interval of Ki67-Count < 40%. Potential clinical impact of the prediction was tested by dichotomising the cases at the cut-off values of 10, 15, and 20%. Misclassification rate of 5-7% was achieved, compared to that of 11-18% for the VE-median-based prediction.

**Conclusions:**

Our experiments provide methodology to achieve accurate Ki67-LI estimation by DIA, based on proper validation, calibration, and measurement error correction procedures, guided by quantified bias from reference values obtained by stereology grid count. This basic validation step is an important prerequisite for high-throughput automated DIA applications to investigate tissue heterogeneity and clinical utility aspects of Ki67 and other immunohistochemistry (IHC) biomarkers.

## Introduction

Rapid development of digital pathology technologies, enabling high-resolution scanning of microscopy slides, brings great efficiencies in data storage, transfer and usage in research, clinical practice and education [1-3]. The most unique and significant benefit for pathology practice and research can be expected from digital image analysis (DIA) applications, opening new perspectives for pathology to serve the needs of personalized medicine, by providing more accurate and reproducible measurements for tissue-based diagnosis, prognosis and prediction [4,5]. Microscopic images, used in pathology, contain an enormous amount of data that can be retrieved by numerous methods available to visualise tissue, cell and molecular components, scan and process the images, generating rich multi-parametric data of broad dynamic range. In a broader context of biology, the quest for quantitative microscopy, with support of bio-image informatics, raises the perspective that the days of manually chosen “representative” images are numbered and such images will be replaced by quantitative measures based on the underlying image data [6]. Similarly, pathology is becoming a quantitative or analytical discipline and has to adopt both benefits and obligations that come together [7].

The most immediate benefits of DIA come with increased capacity, precision and accuracy, compared to visual evaluation or counting, used in pathology diagnosis and research. While the capacity and precision (reproducibility and repeatability) aspects are rather obvious, the concept of accuracy (objectivity, correspondence to ground truth, criterion standard or reference values) is less familiar to anatomic pathologists and is frequently confused with the reproducibility aspect. This is probably due to the fact that anatomic pathology has been a qualitative and semi-quantitative discipline for many years, while pathology diagnosis itself was seen as the ground truth in medicine. Therefore, reproducibility rather than accuracy of pathology diagnosis or evaluation was mostly the focus. On the other hand, targeted therapies should be validated against and along with specific biomarker tests, leading to the development of standard testing procedures and clinically validated cut-off values. The validated tests and therapies are considered clinically useful; however, usefulness should not become a substitute for accuracy or objectivity [8].

Standardization of DIA for optimal use in pathology involves many aspects - from tissue processing, sampling, staining, scanning, to DIA settings and proper test validation requirements, as extensively reviewed [8,9]. Although no studies have performed a full scale investigation of every aspect of the DIA process, the combined evidence shows that DIA is able to reproduce data at an acceptable level, with no more variability than manual assessment using conventional microscopy. Meanwhile, validation of DIA has been performed by comparing digital results with manual estimates, either quantitative or semi-quantitative, or by comparing DIA with another form of criterion standard, for example, fluorescence *in situ* hybridization, or by comparing DIA with clinical (often prognostic) information [9].

Although these validation approaches are common and useful, a criterion standard in these studies is still indirect and may be subject to its own bias. Ideally, to validate and calibrate the DIA tools one should seek the most direct reference values (RV) that answer the same question as the algorithm is intended to do [7]. This means that the same feature in the same image has to be measured by an independent and most possibly objective way; therefore, stereologically sound methods have to be re-introduced to serve the validation and quality assurance of DIA tools; in other words, the DIA tools have to produce stereologically valid results [7,9].

Most useful DIA applications in pathology can be expected today in the area of immunohistochemistry (IHC), a widely-used and relatively inexpensive technology, enabling a broad spectrum of tissue-based biomarkers for personalized therapies; therefore, raising requirements for IHC quantification and accuracy. Not surprisingly, many DIA studies have been targeting IHC markers in breast cancer and other pioneering areas of personalized therapies. As an example, a paradox of an outstanding issue of the cell proliferation marker Ki67 in breast (and other) cancers can be recognized: it is regarded as an important prognostic and predictive factor; however, its clinical utility is hindered by the absence of harmonized methodology of the test [10,11]. Besides the need for accurate enumeration of the proportion of Ki67-positive tumour cell profiles (Ki67 labelling index - Ki67 LI), the issue is further complicated by marked intra-tumour heterogeneity of Ki67 expression in many cases, therefore, demanding standardized sampling of the tissue for the analysis. Although DIA is welcomed, current clinical recommendation asks pathologist to score at least 1,000 cells while 500 cells would be acceptable as the absolute minimum [11].

Gudlaugsson *et al*. [12] have recently compared the reproducibility and prognosis prediction accuracy of different techniques for measurement of Ki67 LI in breast cancer. Two pathologists performed global subjective impression assessment of Ki67 positivity by rapidly scanning/estimating the percentage of Ki67-positive nuclear profiles. Secondly, accurate subjective counts were performed by first identifying hot-spots of Ki67 expression on a whole section at low magnification; in the hot-spot with the subjectively highest Ki67 expression, the Ki67 LI was assessed by two pathologists independently. The third method involved computerized interactive morphometric (CIM) assessment to overcome selection bias. Finally, the DIA was performed on 2 to 10 square areas with the subjectively estimated highest Ki67 LI. The authors concluded that Ki67 LI by DIA, but not subjective counts, was reproducible and prognostically strong. The CIM was also highly reproducible between the two pathologists, but no direct (image-based) comparison of the CIM and DIA was stated in this report.

We concur with the notions that validation of DIA tools is a multi-step process to consider all potential sources of variation. Presuming that pre- and analytical IHC variation needs to be dealt with by routine quality assurance processes, the DIA methods add unique processes of slide scanning, region of interest selection, object segmentation, characterization, enumeration and evaluation. Yet, it is hardly possible to properly address all aspects in one study. With the aim to develop a sound DIA validation and calibration methodology, we designed our experiment to test and improve the accuracy of Ki67 LI estimation by automated DIA on preselected tissue microarray (TMA) Ki67 IHC images, with the ground truth obtained by counting tumour cell profiles using a stereology test grid of systematically sampled frames. We therefore minimized the impact of the tissue heterogeneity and IHC variability, aspects to be addressed separately. In addition, we evaluated the accuracy of visual assessment (impression) of five pathologists on the same images, to simulate the widely used practices to test DIA results against visual estimates or their averaged values.

## Materials and methods

### Population

This study was performed on TMA images from 164 female patients with an invasive ductal carcinoma of the breast, treated at the Oncology Institute of Vilnius University and investigated at the National Center of Pathology, during the period of 2007 to 2009. The study was approved by the Lithuanian Bioethics Committee. The patients’ consent to participate in the study was obtained.

### Tissue preparation

The TMAs were constructed, stained and scanned as described previously [13]. Briefly, one millimetre-diameter cores were punched from tumour areas randomly selected by the pathologist and paraffin sections were cut at 3 μm-thickness.

### Immunohistochemistry (IHC)

IHC for Ki67 was performed with a multimer-technology based detection system, ultraView Universal DAB (Ventana, Tucson, AZ, USA). The Ki67 antibody (clone MIB-1; DAKO, Glostrup, DK) was applied at a 1:200 dilution for 32 minutes, followed by the Ventana BenchMark XT automated immunostainer (Ventana) standard Cell Conditioner 1 (CC1, a proprietary buffer) at 95°C for 64 minutes. Finally, the sections were developed in DAB at 37°C for eight minutes, counterstained with Mayer’s hematoxylin and mounted.

### Image acquisition

Digital images were captured using the Aperio ScanScope XT Slide Scanner (Aperio Technologies, Vista, CA, USA) under 20x objective magnification (0.5 μm resolution). One TMA spot image per patient was used for the study.

### Quantification with stereology test grid

**Figure 1:**
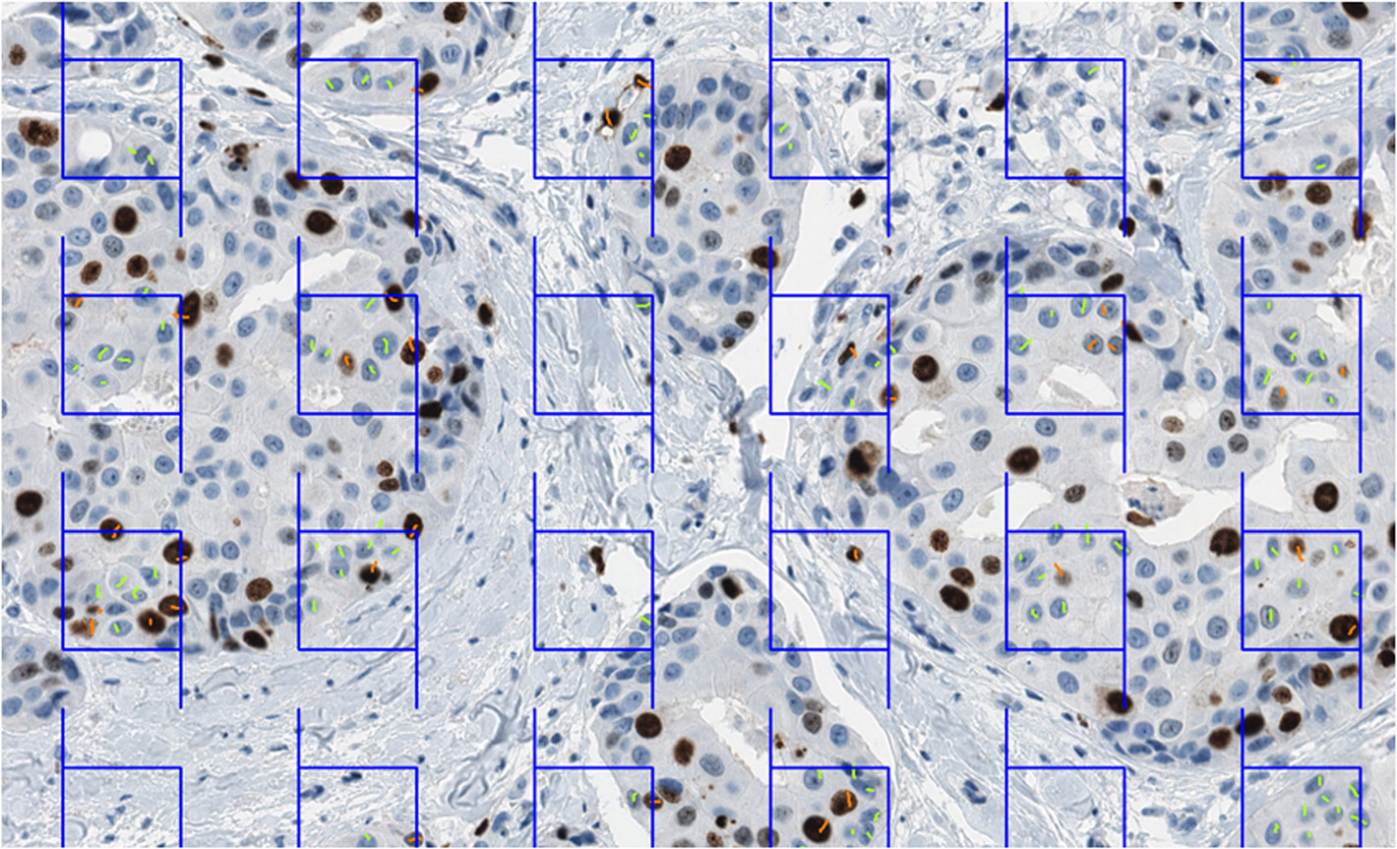
**Test grid of frames from the stereology module overlaid on the TMA spot image.** The left and bottom lines of a frame are “forbidden” - nuclear profiles intersecting them are not marked. The short line marks (orange for Ki67-positive, green for Ki67-negative tumour cell nuclear profiles) are produced manually by an observer. Total numbers and Ki67 LI are computed by the software at the end of the procedure. TMA, tissue microarray.

**Figure 2:**
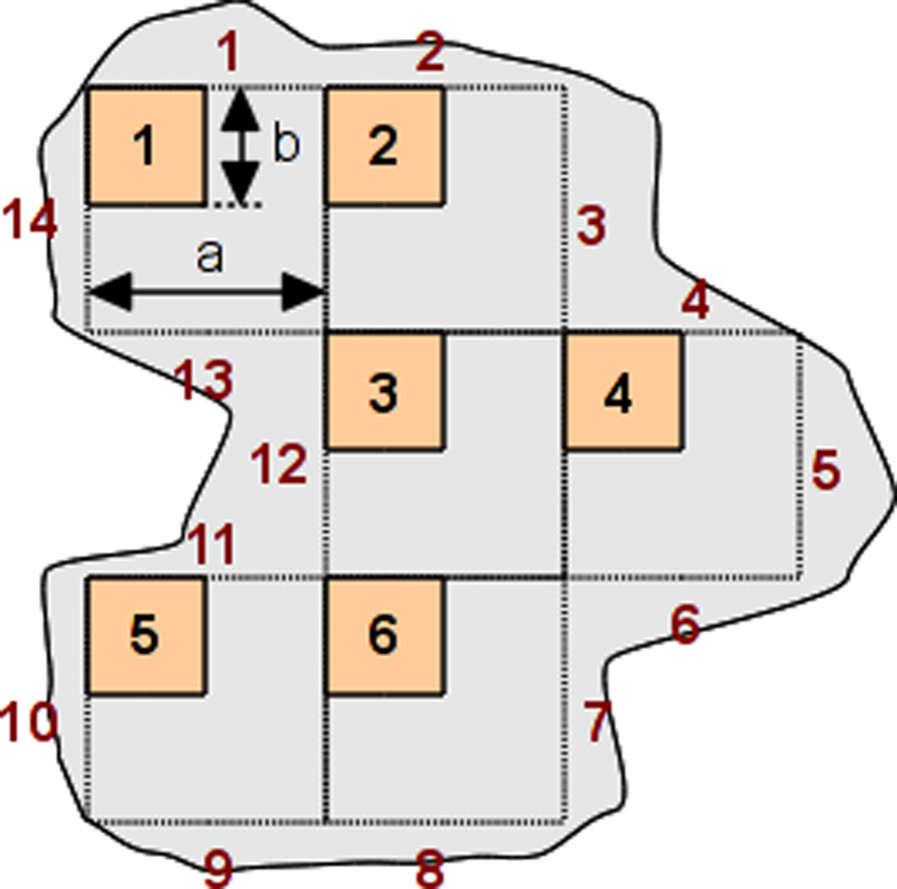
**Tumour area (grey) and test grid of systematically sampled frames (orange) (a = 250 pixels, b = 125 pixels).** For this example, the number of frames is n = 6 and the number of external segments is m = 14.

RV were obtained by marking Ki67-positive and negative tumour cell profiles, using a stereological method for 2D object enumeration [14,15] implemented by the Stereology module (ADCIS, Caen, France) with a test grid of systematically sampled frames (frame size - 125 pixels, spacing of frames - 250 pixels) overlaid on a spot image in ImageScope (Aperio Technologies, USA), Figure 1. The percentage of Ki67 positive tumour cell profiles established by the test grid estimation (Ki67-Count) was calculated as 100*Ki67-positive nuclear profiles/(Ki67-positive nuclear profiles + Ki67-negative nuclear profiles). To test the degree of uncertainty of the RV, inter-observer variation was estimated based on Ki67-Count values produced by three observers (Ki67-Count-1, 2 and 3) independently in a subset (n = 30) of the TMA images. Since the inter-observer variability was found to be negligible (see Results), the RV in the whole series (n = 164) were established by one-observer marking (Ki67-Count), splitting the job among four observers in approximately equal proportions. Estimated time to produce cell marks on the frame grid was 30 minutes per one TMA spot image on average but varied due to variable cellularity of the tumour tissue. Also, the uncertainty of the RV was estimated through Coefficient Error (CE) computation, according to the sampling theory [16]: this uncertainty originates from the fact that the frame count is performed on the subsampled tissue and is calculated as CE = t.sqrt(Cg.(m/n^2^)) [17] with n being the number of frames inside the tumour, m being the number of the external sides of the set of frames in the tumour. For the test grid (Figure 2) with a frame size of 125 pixels and a frame spacing of 250 pixels, the value of the grid factor Cg is 0.049. Otherwise, the value of the Student factor *t* is 2 for a confidence of 95% and for an event number greater than 30.

### Visual evaluation (VE)

A global subjective impression for the Ki67 LI on the same images was performed by five pathologists independently and provided semi-quantitative values (Ki67-VE-1, 2, 3, 4 and 5) expressed as the percentage of Ki67-positive tumour cell profiles. Counting was not included in the procedure.

### Digital Image Analysis

DIA was performed with Aperio Genie and Nuclear v9 algorithms enabling automated selection of the tumour tissue (the Genie Classifier was trained to recognize tumour tissue, stroma and background (glass), then combined with the Nuclear algorithm). Several calibration cycles of the DIA (named DIA-0, 1 and 2, resulting in the percentage of Ki67-positive tumour cells - Ki67-DIA-0, 1 and 2, respectively) were performed to improve the accuracy of the tool by adjusting the settings of the Nuclear algorithm (Table 1). Ki67-DIA-0 was obtained by the default Aperio settings for the Nuclear algorithm, Ki67-DIA-1 - by “subjective” visual assessment of the quality of the DIA results on the computer monitor; Ki67-DIA-2 was fine-tuned based on the quantitative bias established by statistical analyses comparing the Ki67-DIA-1 to RV (Ki67-Count). Highly automated calibration cycles were achieved by developing software to integrate the DIA outputs and statistical analysis procedures.

**Table 1:** Nuclear algorithm settings for the DIA calibration after the Genie classifier

**Algorithm setting**	**DIA-0**	**DIA-1**	**DIA-2**
Averaging radius (μ)	1	1	1
Curvature threshold	2.5	2.5	2.5
Segmentation type	Cytoplasm rejection	Cytoplasm rejection	Cytoplasm rejection
Threshold type	Edge threshold	Edge threshold	Edge threshold
Lower intensity threshold	0	0	0
**Upper intensity threshold**	**220**	**230**	**230**
**Min. nuclear size (**μ^2^**)**	**20**	**45**	**40**
**Max. nuclear size (**μ^2^**)**	**1,000,000**	**1,000**	**1,000**
Min, roundness	0.1	0.1	0.1
**Min. compactness**	**0**	**0**	**0.2**
**Min. elongation**	**0.1**	**0.1**	**0.2**
Remove light objects	removes no nuclei	removes no nuclei	removes no nuclei
**Weak (1+) threshold**	**210**	**210**	**229**
Moderate (2+) threshold	188	188	188
Strong (3+) threshold	162	162	162
Black threshold	0	0	0
Edge trimming	Weighted	Weighted	Weighted

DIA-0 (default), DIA-1 (subjective) and DIA-2 (based on quantified bias). Modified settings are highlighted in bold.

### Statistical analysis

Accuracy of the DIA and VE with regard to the RV was estimated by one-way ANOVA (Duncan multiple range test was used for pairwise comparisons), Pearson correlation, single and multiple linear regression analyses, as well as orthogonal linear regression based on principal component analysis. Agreement between individual measurements was also estimated based on 95% confidence intervals calculated from the RV CE and visualized by Bland and Altman plots [18]. Dependence of RV (n = 30) and VE (n = 164) inter-observer variation on the magnitude of measurement was visualized by plots of corresponding standard deviations against the mean values of the measurements. A variable degree of right asymmetry (skewness from 0.5 to 1.6) of the parameter distribution was noted; where appropriate, statistical significance of the findings was verified, using log-transformed data. Statistical significance level was set at *P* <0.05. Statistical analyses were performed with SAS 9.3 software, Microsoft Excel software (Microsoft, Redmond, Washington, USA) and OpenOffice Calc software (Oracle, Redwood City, California, USA).

## Results

### Characteristics and measurement uncertainty of the reference value dataset

Summary statistics of the RV (n = 30) obtained by three independent observers’ marking of the tumour cell profiles in the test grid are presented in Table 2, along with the results of other measurements in this dataset for reference. No significant variance between the three Ki67-Counts was revealed by one-way ANOVA (F = 0.08, *P* = 0.9217), while strong pairwise correlation among the values was found: r = 0.98, r = 0.98, r = 0.97 (*P* <0.0001). Similarly, the total number of nuclear profiles marked did not differ significantly, although Observer 1 tended to mark less; the total number of nuclear profiles of Observer 1 correlated with that of observers 2 and 3 at r = 0.94, while the latter two correlated at r = 0.98 (*P* <0.0001).

**Table 2:** Summary statistics of the reference values produced by observers, visual estimates and image analyses (n = 30)

**Variable**	**Median**	**Mean**	**Std dev**	**Std error**	**Min**	**Max**
Ki67-Count-1	21.7	28.6	20.4	3.7	0.3	72.6
Ki67-Count-2	24	29.9	19.5	3.6	0.6	69.7
Ki67-Count-3	23	28.7	18.6	3.4	1.2	69.4
Ki67-Count-median	24	29.3	19.4	3.5	0.6	67.4
Ki67-Count-mean	23.4	29.1	19.4	3.5	0.7	66.8
Total profiles Observer 1	331	425.7	273.7	50	85	1,098
Total profiles Observer 2	509	590.7	385.4	70.4	143	1,863
Total profiles Observer 3	471.5	547.2	331.9	60.6	146	1,544
Ki67-VE-1	10	18.3	15.3	2.8	5	70
Ki67-VE-2	30	40.2	29.4	5.4	2	95
Ki67-VE-3	37.5	41.4	27.7	5.1	1	90
Ki67-VE-4	20	30.2	23	4.2	4	80
Ki67-VE-5	22.5	31	24.1	4.4	1	90
Ki67-VE-median	22.5	32.5	25	4.6	2	90
Ki67-VE-mean	23.4	32.2	23.2	4.2	6.2	80
Ki67-DIA-0	16.1	19.9	12.5	2.3	2.1	50
Ki67-DIA-1	18.5	24.8	15.9	2.9	1.6	65.5
Ki67-DIA-2	22.8	29.1	15.7	2.9	9.1	68.4

**Figure 3:**
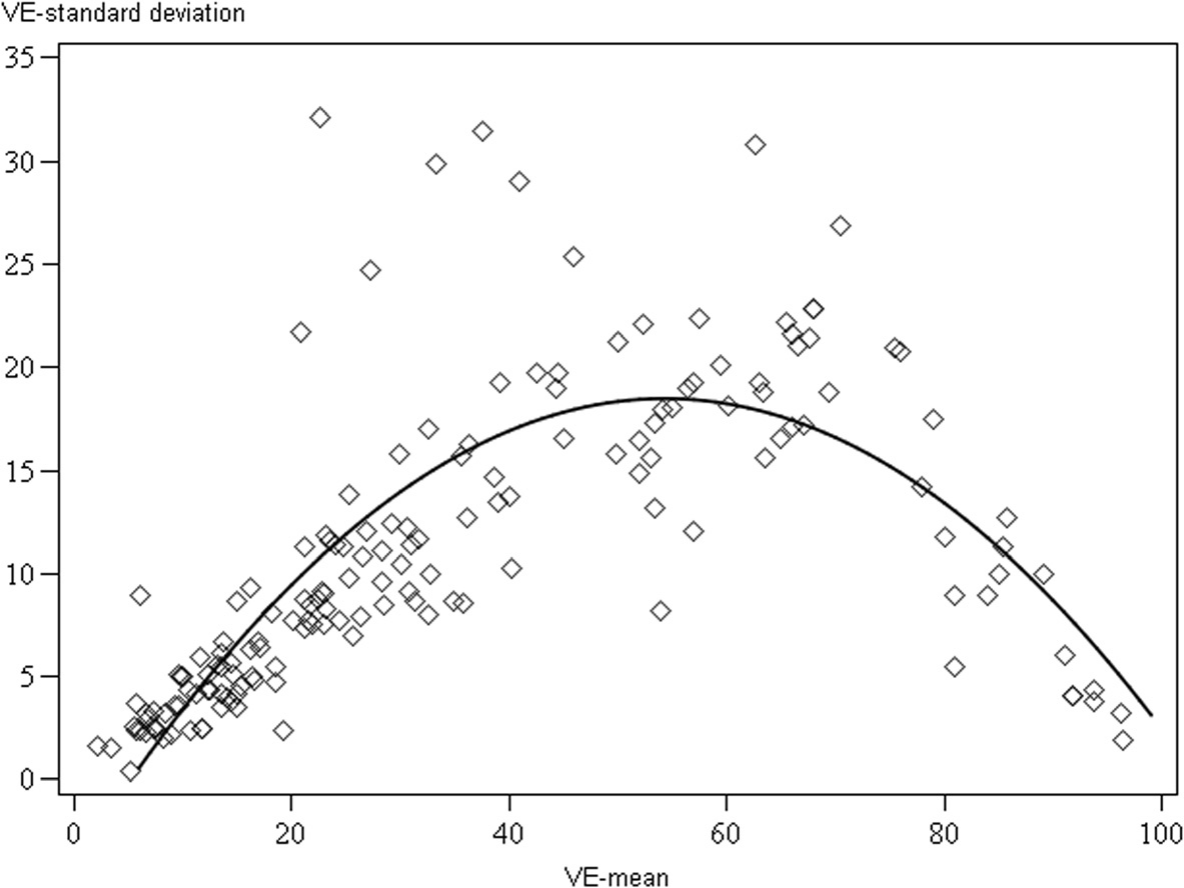
A scatter plot of the means and standard deviations of the five pathologists’ visual estimates (n = 164) with interpolation line.

Uncertainty introduced by variance among the three counts to produce Ki67-Count for each individual spot was low: for the 30 spots, mean standard deviation and mean standard error were 2.6% and 1.5%, respectively. Of note, the five visual estimates (Ki67-VE), summarized for the same individual 30 spots, revealed much higher uncertainty - mean standard deviation and mean standard error were 10.9% and 4.9%, respectively. Interestingly, a scatter plot of the five VE’ standard deviations against their means (n = 164, Figure 3) uncovered nonlinear relationship reflecting higher variation in the middle of the means’ scale. Meanwhile, the positive linear relationship between the standard deviations and the means for the three observers of Ki67-Count was found in the dataset available (n = 30, not shown).

**Figure 4:**
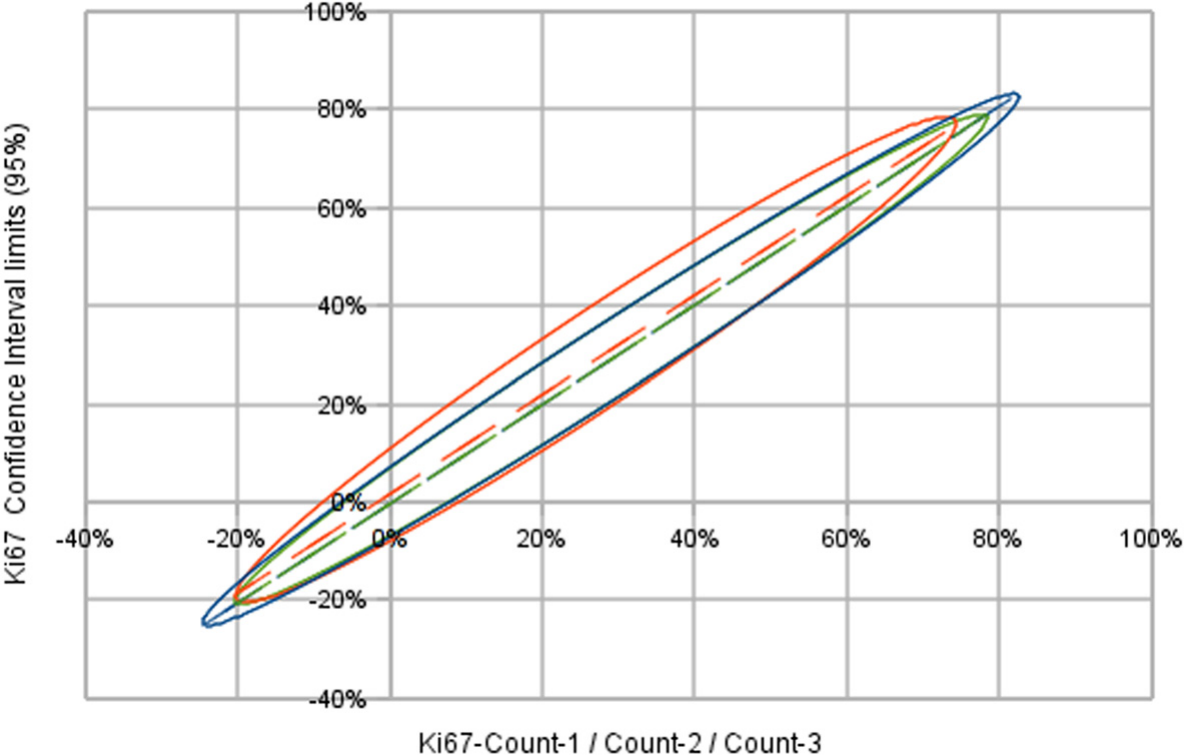
**The ellipses computed from the limits of the confidence interval (CI 95%) for the three independent Ki67 counts (n = 30).** Observer ellipses are almost superimposed: Ki67-Count-1 limit is the blue ellipse (centre x = 29%, y = 29%; major axis = 76%; minor axis = 6%; tilt = 45.36°); Ki67-Count-2 limit is the orange ellipse (centre x = 27%, y = 29%; major axis = 68%; minor axis = 8%; tilt = 46.38°); Ki67-Count-3 limit is the green ellipse (centre x = 29%, y = 29%; major axis = 70%; minor axis = 6%; tilt = 45.37°).

Uncertainty caused by subsampling the tissue by the test grid of frames was estimated by computation of CE providing confidence intervals for each individual Ki67-Count value. Overlap of the Ki67-Count confidence intervals for all three and each pair of the three observers was considered as agreement between the generated Ki67 LI values (Figure 4). The agreement within the same confidence interval among all three measurements was 69%; whereas the pairwise agreement varied from 83 to 86%. The uncertainty of the RV generated was therefore considered satisfactory. The RV for the whole image dataset (n = 164) were based on a single observer count per spot (Ki67-Count). Yet, the subsampling uncertainty was further taken into account in the accuracy estimates.

### Accuracy of the image analysis and visual estimates with regard to the reference values

**Figure 5:**
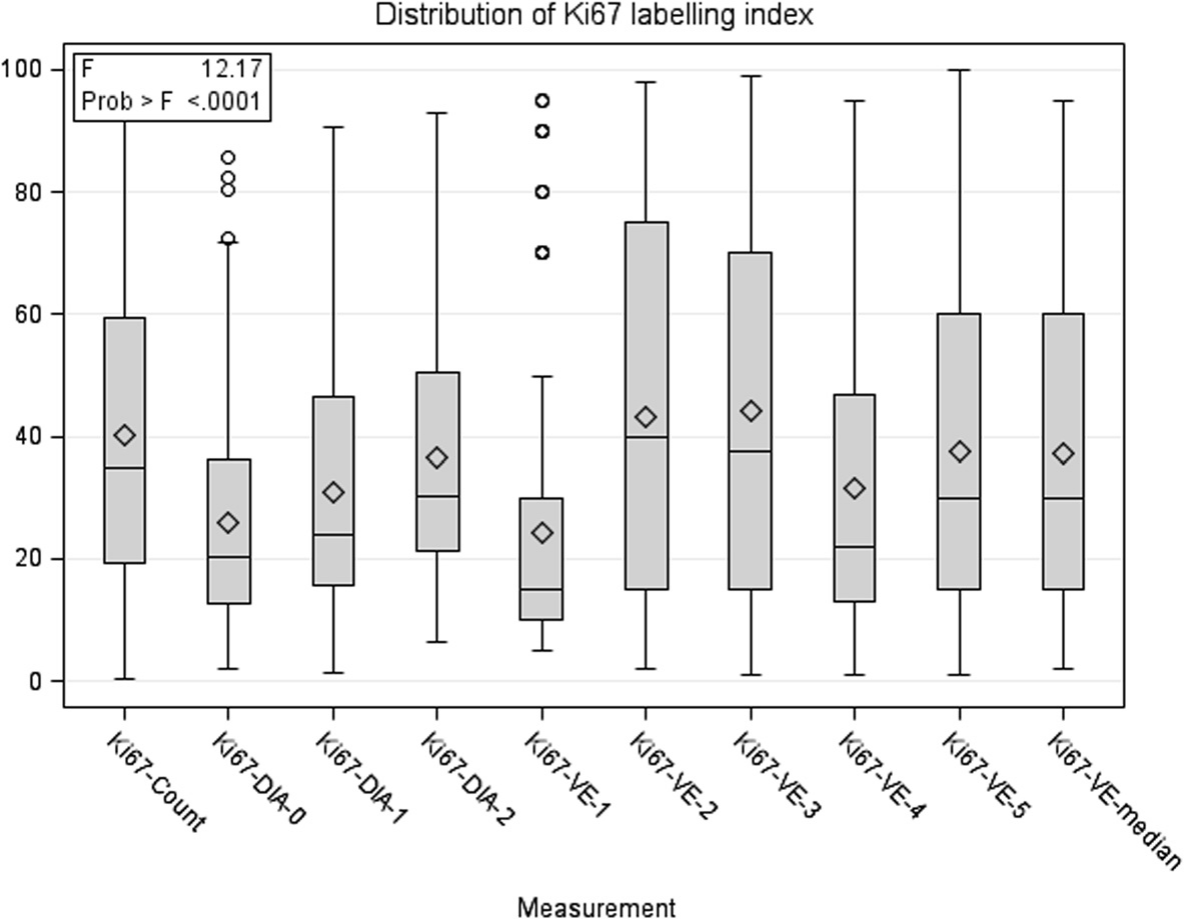
One-way ANOVA box and whisker plot of the distribution of Ki67 labelling index by the method of measurement (n = 164).

Summary statistics of the RV, DIA and VE variables (n = 164) are presented in Table 3. One-way ANOVA revealed significant variance explained by the measurement method overall (Figure 5, *P* <0.0001). Pairwise comparisons (Table 4) revealed no significant bias among the Ki67-Count and Ki67-VE-2 and Ki67-VE-3 estimates (Duncan grouping A) or Ki67-VE-5, Ki67-VE-median and Ki67-DIA-2 (Duncan grouping B). Meanwhile, Ki67-DIA-0, Ki67-DIA-1, Ki67-VE-1 and Ki67-VE-4 produced significantly lower values.

**Table 3:** Summary statistics of the reference values produced by three observers with the corresponding data of visual estimates and digital image analysis, n = 164

**Variable**	**Median**	**Mean**	**Std dev**	**Std error**	**Min**	**Max**
Ki67-Count	35.0	40.2	25.3	2.0	0.6	98.1
Ki67-DIA-2	30.1	36.5	20.2	1.6	6.4	93.0
Ki67-DIA-1	24.1	31.1	21.1	1.6	1.5	90.5
Ki67-DIA-0	20.4	25.9	18.1	1.4	2.1	85.7
Visual median	30	37.2	27.4	2.1	2	95
Visual mean	28.4	36.2	25.6	2.0	2.2	96.4
Ki67-VE-1	15	24.3	23.6	1.8	5	95
Ki67-VE-2	40	43.4	29.6	2.3	2	98
Ki67-VE-3	37.5	44.1	30.0	2.3	1	99
Ki67-VE-4	22	31.6	24.3	1.9	1	95
Ki67-VE-5	30	37.7	27.7	2.2	1	100
Total profiles observer*	2,372	2,658.7	1,390.4	108.6	464	7,452
Total profiles DIA-2	2,150.5	2,293.2	796.8	62.2	752	4,302
Total profiles DIA-1	1,920.5	2,022.7	670.1	52.3	1,012	3,788
Total profiles DIA-0	4,203.5	4,385.0	1,420.2	110.9	1,640	7,939

*Total nuclear profiles observer counts are multiplied by four in this table to be comparable to the DIA total profile numbers (the box grid used for the observer count covers ne-fourth of the image area). DIA, digital image analysis; VE, visual estimate.

**Table 4:** Pairwise comparisons for the means of reference values, visual estimates and digital image analysis results, n = 164

Duncan grouping*			**Mean**	**Measurement**
	A		44.1	Ki67-VE-3
	A		43.4	Ki67-VE-2
B	A		40.2	Ki67-Count
B			37.7	Ki67-VE-5
B	C		37.2	Ki67-VE-mean
B	C	D	36.5	Ki67-DIA-2
	C	D	31.6	Ki67-VE-4
	E	D	31.1	Ki67-DIA-1
F	E		25.9	Ki67-DIA-0
F			24.3	Ki67-VE-1

*Means with the same letter are not significantly different at *P* <0.05. DIA, digital image analysis; VE, visual estimate.

Pairwise correlations (Table 5) were highly significant (*P* <0.0001). Remarkably, correlation between Ki67-Count and Ki67-DIA-0, 1 and 2 improved which each calibration cycle from r = 0.928 to r = 0.949. Notably, Ki67-Count correlated with the Ki67-VE-median strongest (r = 0.930), in comparison to the correlations with the individual VE measurements.

**Table 5:** **Pairwise correlations between the reference values, visual estimates and digital image analysis results (Pearson’s coefficients, *P *** **<0.0001, n = 164)**

**Measurement**	**Ki67-count**	**Ki67-DIA-2**	**Ki67-DIA-1**	**Ki67-DIA-0**	**Visual median**	**Ki67-VE-1**	**Ki67-VE-2**	**Ki67-VE-3**	**Ki67-VE-4**
Ki67-DIA-2	0.949								
Ki67-DIA-1	0.945	0.989							
Ki67-DIA-0	0.928	0.974	0.976						
Ki67-VE-median	0.930	0.940	0.946	0.927					
Ki67-VE-1	0.861	0.917	0.921	0.925	0.891				
Ki67-VE-2	0.905	0.905	0.915	0.886	0.955	0.829			
Ki67-VE-3	0.921	0.921	0.931	0.900	0.969	0.857	0.972		
Ki67-VE-4	0.887	0.894	0.895	0.884	0.936	0.857	0.881	0.901	
Ki67-VE-5	0.842	0.869	0.872	0.860	0.916	0.822	0.853	0.872	0.829

DIA, digital image analysis; VE, visual estimate.

Single linear regression analyses for the DIA and VE results as dependent variables and the RV as explanatory variables produced highly significant (*P* <0.0001) models in all cases (Table 6). Remarkably, determination coefficients (R-square) improved with each calibration cycle of the Ki67-DIA-0, 1, and 2 from 0.86 to 0.89 and 0.90. Notably, R-square for the VE-median (0.86) was the highest amongst the individual VE but reached only that of the Ki67-DIA-0.

**Table 6:** **Single linear regression models with reference values as explanatory variable (n = 164, *P *** **<0.0001 for all models and slope estimates)**

**Variable**	**R-square**	**Intercept estimate**	**Intercept** ***P***	**Slope estimate**	**Slope standardized estimate**
Ki67-DIA-2	0.90	5.9692	<0.0001	0.7588	0.9494
Ki67-DIA-1	0.89	-0.6389	0.5324	0.7892	0.9447
Ki67-DIA-0	0.86	-0.9576	0.3389	0.6667	0.9278
Ki67-VE-median	0.86	-3.3799	0.0242	1.0093	0.9316
Ki67-VE-1	0.74	-8.1114	<0.0001	0.8057	0.8514
Ki67-VE-2	0.82	0.6733	0.7180	1.0616	0.9049
Ki67-VE-3	0.85	0.1337	0.9382	1.0926	0.9210
Ki67-VE-4	0.79	-2.7516	0.0987	0.8545	0.8545
Ki67-VE-5	0.71	0.4763	0.8294	0.9245	0.8422

DIA, digital image analysis; VE, visual estimate.

**Figure 6:**
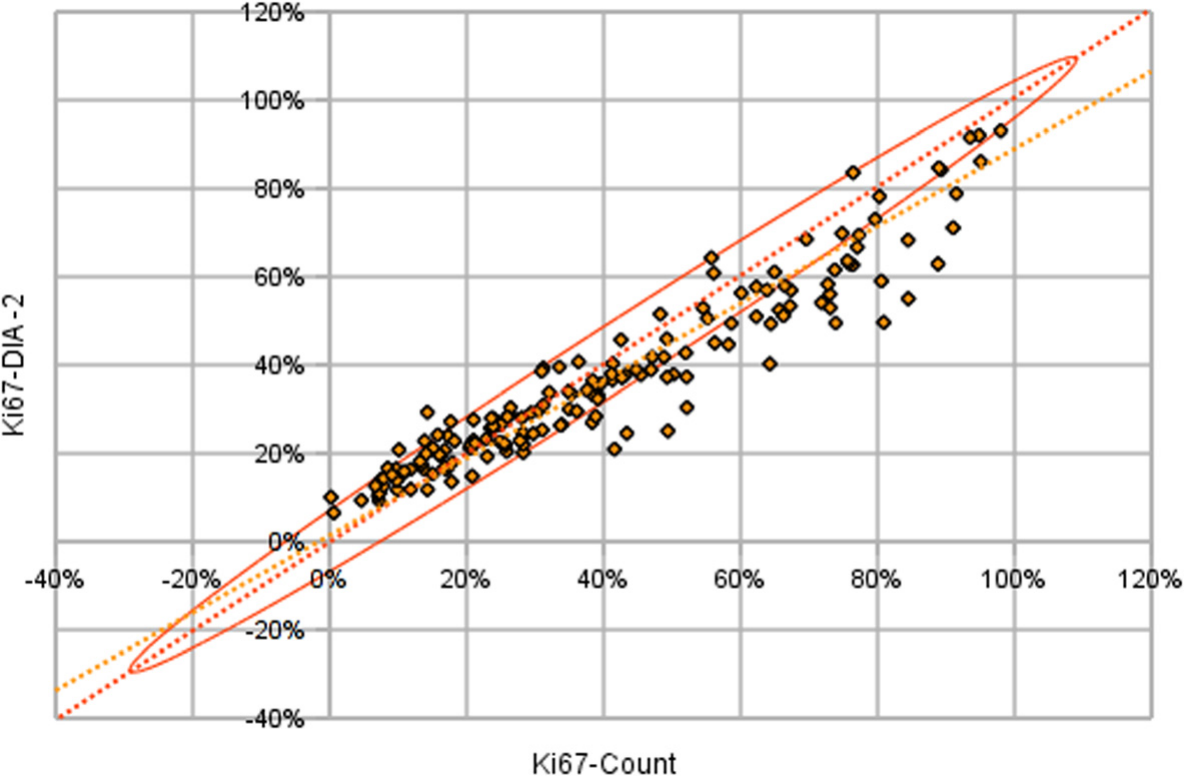
**Orthogonal linear regression analysis.** Reference values as explanatory variable and the DIA-2 as dependent variable (yellow) taking into account an ellipse of 95% confidence interval (orange) defined the sampling theory (n = 164 n = 164, P <0.0001, equation of the line: y = 0.877x + 0.012). DIA, digital image analysis.

The correspondence between the Ki67-DIA-2 and the RV was also tested, taking into account the uncertainty of the RV related to the subsampling of the tissue by the test grid. The confidence interval for the RV was calculated and the Ki67-DIA-2 values were tested for fitting the confidence interval (Figure 4). The R-square of the model was 0.90, the accuracy factor was 0.82. Interpretation of the plot and the slope tilt from the ellipse axis revealed a bias: underestimation of the Ki67-Count by the Ki67-DIA-2 observed at the higher end of the RV scale as well as overestimation at the low end. Similarly, Bland and Altman plots (not shown) reflected the same bidirectional bias dependent on the magnitude of the measurement. Orthogonal linear regression analysis for the DIA and RV was used to refine the accuracy value reducing the intercept factor. In this case, the ratio of the tilt of the regression line and the tilt of the ellipse axis defining the accuracy factor was 0.92 (Figure 6).

Outliers of the Ki67-DIA-2 versus RV analyses were inspected to explore potential reasons of the underestimation. In general, the tumour tissue was highly cellular in many cases resulting in overlapping nuclei and their confluence and/or rejection by maximum size limit at the DIA. Also, in some cases, tissue artefacts, an admixture of stroma with lymphocytes and large ducts could impact the DIA results. Further fine-tuning of the Nuclear algorithm settings was attempted without notable success.

### Prediction of the reference values by inverse regression and measurement error correction

Ki67-DIA-2 enabled fair accuracy and outperformed the 5 VE measurements, both individual and the median. Yet, the measurement bias for the Ki67-DIA-2 was established and enabled a measurement error correction procedure to be used to predict the ground truth in real life with maximum accuracy. Inverse regression analyses were performed to retrieve the correction criteria (Table 7). To avoid the potential impact of some non-linearity noted and to derive the most useful inverse regression model for accurate prediction of the ground truth in the interval of clinical importance, a regression model Ki67-DIA-2 < 40 was produced, based on the observations with Ki67-Count values less than 40% (n = 92).

**Table 7:** **Single and multiple linear inverse regression models to predict reference values as dependent variable (n = 164, *P *** **<0.0001 for all models and slope estimates)**

**Variable**	**R-square**	**Intercept estimate**	**Intercept** ***P***	**Slope estimate**	**Slope standardized estimate**
***Single regression models:***					
Ki67-DIA-2	0.90	-3.1183	0.0165	1.1878	0.9494
Ki67-DIA-2 < 40*	0.75	-4.3913	0.0085	1.1472	0.8688
Ki67-DIA-1	0.89	5.0453	<0.0001	1.1309	0.9447
Ki67-DIA-0	0.86	6.8232	<0.0001	1.2916	0.9278
Ki67-VE-median	0.86	8.3195	<0.0001	0.8572	0.9302
***Multiple regression model***	0.91	-0.3245	0.8096		
Ki67-DIA-2				0.8068	0.6448
Ki67-VE-median				0.2985	0.3239

*Ki67-DIA-2 < 40 - represents a regression model for Ki67-DIA-2 with only Ki67-Count less than 40% cases included in the analysis (n = 92). DIA, digital image analysis; VE, visual estimate.

In addition to the single regression models, multiple regression models with inclusion of both Ki67-DIA-2 and Ki67-VE-Median gave slightly higher R-square value (0.91) than the Ki67-DIA-2 alone (0.90). Therefore, the DIA approach with calibration of the algorithm settings based quantified bias enabled most accurate measurement of the Ki67 LI, while VE of five pathologists were consistent but gave little added value in terms of accuracy, compared to the automated DIA measurement.

### Effect of the prediction and measurement error correction on Ki67 dichotomisation accuracy

The effect of VE and DIA inverse regression models to predict the RV on accuracy of patient dichotomisation at RV cutoffs of clinical importance (>10, 15 and 20%) was tested (Table 8). The cutoffs used for these simulations were the ones most commonly considered as clinically relevant to test potential clinical impact of the measurement methods involved in our study. While Ki67-VE-median tended to underestimate the Ki67-Count-based class at all cutoffs, especially at 20%, the Ki67-DIA-2 prediction overestimated the classes, especially at the lower end (>10%) of the scale. Total misclassification rate at different cutoffs varied from 11 to 18% for the VE-based and 5 to 9% for the DIA-based prediction, respectively.

**Table 8:** Effect of the inverse regression-based prediction and measurement error correction on Ki67 dichotomisation accuracy at various reference value cutoffs (n = 164)

**Method**	**Underestimated (%)**	**Overestimated (%)**	**Total misclassified (%)**
Ki-67 cutoff >10%			
Ki67-VE-median	16/148 (11)	2/16 (13)	18 (11)
Ki67-DIA-2	0/148 (0)	12/16 (75)	12 (7)
Ki67-DIA-2 corrected	0/148 (0)	9/16 (56)	9 (5)
Ki67-DIA-2 corrected <40*	2/148 (1)	6/16 (38)	8 (5)
Ki-67 cutoff >15%			
Ki67-VE-median	22/136 (16)	1/28 (4)	23 (14)
Ki67-DIA-2	2/136 (1)	13/28 (46)	15 (9)
Ki67-DIA-2 corrected	3/136 (2)	11/28 (46)	14 (9)
Ki67-DIA-2 corrected <40*	5/136 (4)	6/28 (21)	11 (7)
Ki-67 cutoff >20%			
Ki67-VE-median	28/123 (23)	1/41 (2)	29 (18)
Ki67-DIA-2	2/123 (2)	9/41 (22)	11 (7)
Ki67-DIA-2 corrected	2/123 (2)	12/41 (29)	14 (9)
Ki67-DIA-2 corrected <40*	6/123 (5)	6/41 (15)	12 (7)

*Ki67-DIA-2 < 40 - represents a regression model for Ki67-DIA-2 with only Ki67-Count less than 40% cases included in the analysis (n = 92). DIA, digital image analysis; VE, visual estimate.

The effect of measurement error correction for the Ki67-DIA-2-based prediction of the RV was tested with the values obtained by the inverse regression formula Ki67-DIA-2-corrected = 1.1878*Ki67-DIA-2-3.1183 and, to minimize potential non-linearity impact for the prediction accuracy, by the formula Ki67-DIA-2-corrected <40 = 1.1472*Ki67-DIA-2 -4.3913 (Tables 7 and 8). The error correction for both prediction models (especially, the Ki67-DIA-2-corrected <40 model) decreased the DIA overestimation effect at the >10% cutoff. While total misclassification rate at different cutoffs for Ki67-DIA-2-corrected remained in the interval of 5 to 9%, the Ki67-DIA-2-corrected <40-based prediction enabled some improvement down to the misclassification rate of 5 to 7%.

In summary, the DIA-based prediction of the RV enabled the classification error rate half of that of the VE-based prediction, it was less than 10% at all cutoffs tested, and could be further improved by the measurement error correction attempts.

## Discussion

Our experiment presents test validation, calibration and measurement error correction methodology that can be successfully applied to ensure and improve accuracy of IHC Ki67 LI estimation by DIA. In essence, in our approach we sought to adopt the principles of analytic test validation for IHC DIA-based enumeration of Ki67 LI with quantification of the measurement bias by comparison to the Ki67 LI obtained on the same images by stereological test grid count as most direct criterion standard. Our first step of the DIA calibration (DIA-0 to DIA-1) was achieved by visual (intuitive) quality assessment of the DIA results on the computer monitor of selected images, while the second (DIA-1 to DIA-2) was based on quantified bias from the criterion standard. Our results show that only after the second (quantitative) calibration step, global bias of the DIA became not significant, while regression analyses revealed gradual improvement of the prediction of the DIA outputs with the calibration steps. Although the calibrated DIA-2 revealed the best accuracy achieved, exceeding that of the VE, nonlinearity was noted with some overestimation bias on the low and underestimation bias at the high end of the scale. We subsequently applied measurement error correction procedures by inverse regression to further enhance the DIA test applicability. Finally, we tested potential clinical impact of the accuracy achieved by applying DIA- and VE-based predictions of Ki67 LI to dichotomize patients (images) by frequently used cut off values at 10, 15 and 20% and found that DIA (after quantitative calibration and measurement error correction) enabled the classification error rate 2x less than that of the VE.

In our study we did not strictly follow the guidelines for analytical test validation [19] since the nature of the subject and the criterion standard (IHC image) used are still different from the analytical test samples used in medicine. First, the uncertainty of our criterion standard was tested by independent measurements by three observers on a subset (n = 30) of images and was considered as satisfactory to further rely on one observer counts. Nevertheless, the inter-observer comparison was image-based but not cell-based, and we realize that human judgment/error is still involved when deciding on individual tumour/non-tumour and positive/negative cells even in this stereologically-based approach. Although some uncertainty of our criterion standard has to be taken into account, our data show that it is more reliable than that of the VE consensus of several pathologists and, therefore, should be used for DIA validation needs. Secondly, we have not tested the repeatability of the tests: it would be beyond reasonable effort to repeat the stereological count manually and less important to repeat (intra-observer) VE, since it was not our main focus to investigate the VE accuracy and precision. The repeatability of the DIA in strictly the same conditions is expected to be perfect, the reproducibility of the DIA involving all phases of the Ki67 LI test as well as its robustness to the IHC staining variation was beyond the scope of this study. Third, we did not validate our DIA prediction accuracy on an independent dataset, since it requires another set of criterion standard data that is planned as an output of our next experiment. Fourth, the DIA validation tests in the present study are based on summarized data per image (Ki67 LI), while more rigid individual cell-based comparisons would provide even more granular information on the performance of the DIA tools.

Our approach uncovered a non-linear bias in the opposite directions depending on the magnitude of the measurement of the Ki67-DIA-2, which could hardly be documented by only the subjective assessment of the DIA accuracy of selected images on a computer monitor. To better understand why DIA-2 overestimated the Ki67 LI at the low and underestimated it at the high ends of the scale, we compared absolute numbers of positive and negative tumour cell profiles detected by the DIA-2 and box grid count (data not shown). We found that with increasing both Ki67 LI and the total number of cell profiles counted, DIA-2 tended to under-detect tumour cells, while this effect was more notable for positive cells. To really explore the sources of the bidirectional bias, one needs to design more sophisticated cell-based quality assurance procedures which would also allow testing the impact of cell density and other features. From our data, we can speculate that increased tumour cellularity with more cell profiles overlapping may impact nuclear segmentation quality, while the impact of the automated tumour tissue segmentation by the Genie algorithm remains to be deciphered. In general, this nonlinearity phenomenon seems to originate from “subject-measurement” interaction, where the measured subject has variable characteristics (tumour cellularity, density, texture, staining, section thickness and so on) and a specific DIA algorithm may handle them with variable success. This further highlights the complexity of the automated DIA approaches and the need of appropriate validation and quality assurance procedures.

Although the VE validation was not the main focus of our study, we observed an interesting nonlinear dependence of inter-observer variation on the magnitude of the measurement: high standard deviations in the five VE observers’ mean were noted in the middle of the Ki67 LI scale. This finding is somewhat unexpected, but still consistent with the observation that IHC biomarker distribution artefacts may be generated by subjective visual scoring [20]. Without going into extended speculations on the potential sources of this variation, we see it as additional evidence that individual VE or “eyeballing” cannot serve as reliable measurement when there is increased clinical demand for quantification accuracy. The “consensus” or median VE of five pathologists ensured better accuracy than individual VE; however, it did not reach that of the calibrated DIA. Furthermore, besides being less accurate, precise and practical for clinical use, multi-observer VE should not be used as a criterion standard method for the DIA validation purposes, because of its greater uncertainty level compared to that of DIA or count-based methods.

The deepening gap between the potential clinical utility of the Ki67 LI and availability of robust measurement methodologies is reflected by the St Gallen 2013 consensus [21]: while the cut off <14% remains in the definition of the Luminal A-like tumours, a majority voted for the threshold of ≥20% to define “high” Ki67 status. Furthermore, a concern about the possible under-treatment of patients with luminal disease who might benefit from chemotherapy, justifies use of a lower (local laboratory specific) cut-off to define Ki-67 “high” or use of multi-gene-expression assay results. This approach would potentially require validation studies with clinical outcomes while the measurement methods remain not standardized. In the situation where one laboratory may serve different oncology units, this would become even less realistic. In addition, it is worth noting that there is a fundamental issue in defining and reproducing Ki67 LI cut-offs with the distribution pattern when the great majority of the hormone-receptor positive breast tumours fall into the Ki67 KI interval between 10 to 20%. Therefore, it is intrinsically difficult to meet the clinical demand for accuracy without measurement methods of established and controlled accuracy, preferably indicating confidence intervals for the values. Even more, combinatorial or multiple IHC biomarker systems may be needed to achieve robust prognostic and predictive indicators [13,22,23].

While manual techniques, including VE and counting, have been shown to be poorly reproducible, even at the level of decision on individual cells [24], the only viable alternative to extract most accurate Ki67 LI by IHC test is further sophistication and standardization of DIA methodologies. They enable greater capacity which also involves counting more cell profiles in more tissue samples, which in turn may lead to better accuracy at the low end of the Ki67 LI scale [25]. The success of the DIA in IHC quantification may be variable and depend both on the DIA tools used and a study design. For breast cancer Ki67 LI measurement, DIA has been shown to be comparable to the VE but of less prognostic value by one study [26] or better than VE, comparable to CIM and of stronger prognostic accuracy by another [12]. Even if it is tempting (and useful) to validate a DIA tool to predict specific clinical outcomes, we argue that sound DIA measurement methods should be developed and maintained by meeting the “basic needs” first to quantify the measurement bias from affordable and most objectively established criterion standard. As put by Bland and Altman [18], “some lack of agreement between different methods of measurement is inevitable, what matters is the amount by which methods disagree”. We, therefore, position our experiment as the first step in DIA validation process to ensure accurate estimation of Ki67 LI in a selected tissue sample, with subsequent steps to use automated DIA to address tissue heterogeneity and sampling issues as well as prediction of clinical outcomes.

## Conclusions

In general, we suggest that proper quantitative validation and calibration methodologies can and have to be employed to establish and ensure accuracy of Ki67 LI measurement by DIA and digital IHC. The measurement accuracy can be further improved by measurement error correction based on the quantified bias, which in our study allowed to decrease patient misclassification rate by the Ki67 LI cut offs of 10, 15 and 20% down to 5 to 7%, compared to that of the VE consensus of five pathologists at 11 to 18%. This basic validation step also opens better perspectives to use high-throughput automated DIA tools to investigate tissue heterogeneity and clinical utility aspects of Ki67 and other IHC biomarker expression.

## Abbreviations

CE: Coefficient error, computed according to the sampling theory; CIM: Computerized interactive morphometric assessment; DIA: Digital image analysis; IHC: Immunohistochemistry; Ki67 LI: Ki67 labelling index; Ki67-Count: Percentage of Ki67 positive tumour cell profiles established by the stereology test grid count; Ki67-DIA: Percentage of Ki67 positive tumour cell profiles established digital image analysis; Ki67-VE: Percentage of Ki67 positive tumour cell profiles obtained by semi-quantitative visual estimate of a pathologist; RV: Reference values; TMA/TMAs: Tissue microarray/tissue microarrays; VE: Visual evaluation.

## Competing interests

The authors declare that they have no competing interests.

## Authors’ contributions

ArL drafted the manuscript, performed statistical analysis, performed stereology count and participated in image analysis calibration experiments. BP drafted essential parts of the manuscript and performed statistical analysis. AiL designed and carried out the image analyses, performed and supervised stereology measurements, and edited the manuscript. PH and NE designed the stereology measurements and edited the manuscript. DD, IB, JB and RM performed stereology measurements. CB, DD, IB and RM performed visual evaluation of the Ki67-LI on the TMA images. YI produced software for TMA image analysis output conversion to streamline statistical analysis cycles and calibration process. IE performed visual evaluation of the Ki67-LI on the TMA images, edited the manuscript and assessed clinical relevance of the findings. All authors participated in conception and design of the study, reviewed the analysis results, and critically revised and approved the final manuscript.
